# Guy’s Hospital Reports (for 1856)

**Published:** 1857-07

**Authors:** 


ART. IV.—*Guy's Hospital Reports.* Edited by SAMUEL WILKS, M.D., LONDIN., and ALFRED POLAND. Third series. Vol. II. London. JOHN CHURCHILL. 1856. 8vo. pp. 416.

IN this familiar and always welcome annual visitant, may be found the usual amount of interesting and practically valuable matter. It belongs to a useful class of foreign additions to our store of knowledge, which cannot be too highly appreciated by all kinds of professional readers. It is obviously more desirable to the genuine American teacher or student, as well as the practitioner, than the best of the annotated text-books of similar origin; and were it likely to be received with the smallest share of the avidity which greets the ordinary reprints, we should be glad to encourage the republication of a work, the influence of which could only be a happy one upon our native literature.

We regard the Guy's Hospital Reports, not only as records and evidences of honorable scientific progress in one of the great centres of medical learning, but as important yearly monitors to those who occupy the posts of responsibility elsewhere—above all, in our own land.

They set us an example, which speaks in tones of unmistakable force to the physicians and surgeons of our metropolitan hospitals; but which we grieve to say these officers have lamentably disregarded.

Neither materials nor qualifications can be wanting in America for the discharge of the duty of clinical reporting in some permanent form; nor do we believe that the support of the profession of the country would be withheld from any properly conducted enterprise, having for its object the systematic cultivation of the boundless fields of observation and experience, which, for any material sign to the contrary, are now lying utterly at waste in most of the large and otherwise admirable clinical charities of our seaboard and inland cities.

A tithe of the industry and intelligence which have been expended, in Philadelphia alone, in the business of introducing other men's ideas and gatherings to the American reader, if devoted to original works, might not merely have added greatly to the already distinguished number and character of indigenous treatises and elementary books, but might long ago have secured to her teachers a pre-eminence in clinical investigation and practical acquirement, which would more than equal the admitted reputation of her rival medical academies. Enough of such legitimate labor has been performed here and in other places, however, to encourage us to look for a development in our native hospital pathology and therapeutics, which shall redeem us from the stigma, only too well deserved, of being without a national school of medical science. Our faith in the vitality of

American medicine is strong, but it sadly needs the aid and comfort of actual fruition.

It was not our intention at this time to dwell upon the theme which has pressed itself almost unavoidably upon us; and we return, therefore, to our proper subject, in a brief analytical commentary upon the work before us.

The first paper of the series gives an account, with an illustration, of a myeloid tumor of the scapula, removed by Mr. Cock from a "remarkably small, delicately made woman," æt. 27, and suckling her third child, an infant six weeks old.

It is chiefly interesting from the careful description given by its author, Dr. Wilks, of the tumor, as a characteristic specimen of a morbid product which has "only of late been fully recognized and described; all growths from bone having been indiscriminately classed under the general name of osteosarcoma."

"The tumor when removed, was found to weigh nearly a pound, and to measure in its long circumference, twelve inches, and in its short, ten inches. It was of an oval shape, having, however, that side concave which constituted its base, and which covered the shoulder. This inferior surface, and the sides immediately springing from it, were bony, so that the greater part of the tumor was enclosed in an osseous shell. The acromion end of the clavicle which had been removed, still articulated with an eminence of bone corresponding to the acromion itself, and the latter was gradually lost in the osseous laminæ which formed the wall of the tumor. It was thus clearly seen, that the growth was derived from an expansion of the spine of the scapula itself, and which, indeed, formed its sides; the most superficial part only of the tumor being soft, and its walls consisting of fibrous membrane.

"Upon making a section of the tumor, a brain-like mass was exposed, enclosed in a thin-walled cyst, the walls being about one-eighth of an inch thick, and bony, with the exception of the most superficial part, which was membranous. Upon closer examination, the internal substance was seen to be composed of an opaque white, curdy structure, and a pink, gelatinous-looking material. The former largely predominated. This was in consistence and color something like cream-cheese, or blancmange, and therefore firmer, drier, and more friable than medullary cancer, and was not capable of emitting any juice, or being formed into a paste by squeezing, as in the latter.

"It was, however, not unlike the appearance presented sometimes by *dead* cancer. The other and characteristic element of the tumor existed only in a minor proportion, and was scattered throughout that just mentioned, in the form of small masses, each about the size of a pea. These were of gelatinous consistence, of a slight translucent appearance, and of a dull pinkish color, this color being altogether peculiar, and one not very often met with either in healthy or morbid structures. The hue was in no way due to blood, for no corpuscles or other elements of this fluid were discoverable. After being in spirit a short time, the color for the most part, disappeared. The whole of the contents of the tumor were very friable, and portions fell out upon everting it.

"*Microscopic examination.*—The white matter was composed principally of fatty granule masses, amongst which were seen the many nucleated cells which composed the entire mass of the translucent portions; from which we conclude that this fat was a superaddition to, or the result of a degeneration of, the other previously existing elements. If this be true, the original structure consisted wholly of the pink semi-transparent parts which were now found to occupy only a minor portion of the tumor; and, therefore, if the latter had been seen at an earlier period, its whole appearance and composition would have had this character. At the time of removal, however, this was being fast destroyed by a fatty degeneration. The elements of which we speak, composing this part, consisted of the mother-cells, described by Lebert as characteristic of one of his forms of fibro-plastic growths, which peculiarity has led Paget, with good reason, to separate those tumors which possess them into another class, which he calls 'myeloid,' a designation founded upon the resemblance of the cells to those existing in the *marrow* of the fœtal bones.

"The cells in this tumor corresponded in most respects with those described by the above-named authors, excepting that the cells were much larger, and contained many more nuclei, some cells holding at least a hundred. Many of the cells, too, were sprouting, sending out processes in all directions, from their sides, which joined and interlocked with each other. It will be seen by the drawing that these cells were of all shapes and sizes, some being small, and others containing only one or two nuclei." (pp. 2–4.)

In answer to the question as to whether this growth is malignant or likely to reappear, the following remarks are important:

"All experience gained at present with respect to these growths, would favor the answer that they do not return,—at least, this is true of the tumor in its pure myeloid form,—the present instance affording a very good example of their history in general, the main points of which are, that they occur in young persons, are isolated, grow from bone, and do not return on removal. The whole question of malignancy, however, is still in abeyance, and appears at present to be only a comparative term, since we continually see simple fibre tissue reproduced after removal, and then acquire the epithet malignant; in the same way osseous growths, from the tendency to develope themselves in a variety of parts, have caused us to recognize a class of diseases known as osteoid cancer, and more lately we have witnessed simple cartilage developing itself in the lymphatic gland, lungs, &c., to the utter destruction of the patient, in the same way as ordinary cancer would have done. The simplest structures, therefore, are not free from the taint of malignancy, using the term in its most general signification. Looking, however, upon myeloid tumors with reference to the question in its older and more restricted sense, we should certainly say that their elements are as benignant as nucleated fibre, bone, cartilage, &c., to which, indeed, they are closely allied. In all probability, however, the harmless and the more destructive elements may be combined; for, as bone may be developed together with cancer, as is seen in osteoid growths, and as cartilage may be developed with cancer, as has been seen in the testis, so, no doubt, myeloid matter may coexist with cancerous elements." (pp. 4, 5.)

Next in order comes a valuable article, embracing the third septennial report of Guy's Hospital Lying-in Charity, from October 1, 1847, to October 1, 1854; and the report of the Lying-in Charity for twenty-one years, from October 1, 1833, to October 1, 1854.

We have in these reports a collation from the records of a very large obstetric clinic, in which the patients are attended at their own houses. The number of cases recorded in the first report is 11,224; in the second it is 22,498. The statistical information is correspondingly abundant, and is presented in every variety of form; while many of the more striking cases and points of practice are given at considerable length, and must prove extremely interesting to the obstetric reader.

The third paper, by Dr. H. M. Hughes, is devoted to, 1, cases of black urine in patients who had been taking creasote; 2, a case of fatal intussusceptio associated with lumbrici; and 3, a case of emphysema of the abdominal parietes from perforation of the rectum.

Black urine, as a sequel of the administration of creasote, according to Dr. Hughes, appears to have attracted the attention of Dr. Elliotson, and was pointed out by him in his paper (Medico-Chirurg. Trans., vol. xix, p. 237) on the medicinal properties of creasote. Dr. Hughes refers, also, to Pereira (Mat. Med.), who quotes some cases by Dr. McCleod, from the Med. Gaz. (vol. ii, p. 599), in which black urine followed the use of creasote, at that time a new remedy. Setting aside three of the cases, as not sufficiently decided, Dr. Hughes signalizes seven undoubted instances "in which the urine was of brownish or purplish-black color."

"In three of these cases creasote had been administered internally; of which in two it had been given in doses of from five to twelve, and even fifteen drops, three times daily; but in the other in a dose of only one drop four times a day; and when it is recollected, how very often this remedy has been administered in the hospital in doses equally large or larger, this effect must be considered as remarkable. In two cases tar had been applied externally for psoriasis, and creasote was obtained from the urine by evaporation; while in the case of the infant related by Dr. Marcet, it is highly improbable that it or any similar substance had been taken; and in the case of the little girl mentioned by myself, it assuredly had not been prescribed. In six of the seven cases the urine was passed black and clear, and remained unaffected, or but slightly affected, by chemical reagents, including heat and nitric acid; whereas, in the seventh case, it was passed of a natural appearance, and only threw down a dense black precipitate upon the addition of nitric acid and the application of heat. Dr. Odling, who examined this urine, states, 'that the black precipitate, on subsequent exposure, became an indigo-blue deposit.'" (p. 58.)

In a postscript to the paper, we are informed of another patient under the reporter's care, in whom the urine became black when boiled with nitric acid. In this, and the other cases where iodine had been taken, the change of color was attributed to the iodine, this metal being supposed to produce the peculiar effect "under certain conditions of disease."

Following these cases, we have a full report, with a lithographic illustration, of the case of intussusception. An unusual form of internal strangulation is in this place very clearly and carefully described from the appearances, on examination, after death.

The paper of Dr. Hughes then terminates with the history of a case of abdominal emphysema from perforation of the rectum, occurring accidentally in a patient affected with cancer of the stomach.

We quote the concluding paragraph, especially on account of the lesson it conveys. The unfortunate accident of which the patient was the victim, was the result of his own mismanagement on this occasion, but is none the less worthy of attention, since, as the reporter justly tells us, it has occurred under other more objectionable circumstances. We know of one instance in which it happened in the hands of a really skilful and experienced surgeon.

"The sequence of events in this case appears to be so clear as not to admit of any doubt. The patient, in the act of passing an enema-tube, experiences great, indeed excruciating pain; fifteen hours afterwards he has emphysema of the integuments of the abdomen; a few days subsequently he has offensive purulent discharges, and a portion of sloughing membrane passed per anum; and after death a large ulcer of the mucous membrane of the rectum is discovered, together with a perforation of the muscular coat. It appeared probable that a large abscess had formed between the mucous and muscular coats; that the former had sloughed away, leaving the latter bare, while the cellular membrane behind it had been comparatively little affected. I believe that cases of perforation of the rectum from the unskilful introduction of clyster-pipes are not very rare, and that most museums contain specimens of the accident, but I do not recollect myself to have met with an instance in which it was productive of emphysema.' (p. 68.)

The paper next in order is a very valuable statistical one, "On Hernia, with an Analysis of 126 Fatal Cases," by Thomas Bryant.

It is intended to aid in the solution of the questions, "What form of hernia is most common? What form most frequently requires operation? And which is most fatal?"

The statistics of the London Truss Society, ending with the year 1855, show that "out of 84,478 cases of inguinal and femoral hernia, 75,054 belonged to the former, and 9424 to the latter division; or, as it is more clearly stated in percentages, out of every hundred cases, 88·8 per cent.

were inguinal, and 11·1 femoral; inguinal hernia being most frequent by 77·7 per cent." Towards the solution of the second query, "281 cases have been collected; of these, 105 were inguinal, and 176 femoral; or, in every hundred cases 37·4 were of the former kind, and 62·6 of the latter. Femoral hernia, therefore, required operation 25·2 per cent. more frequently than inguinal."

In answer to the third question, two tables are presented—one of successful cases, and one of fatal.

"In 169 *cures* after operation, inguinal bore the proportion to femoral as 36·1 per cent. to 62·8, the difference being 27·7 per cent. *in favor* of femoral hernia."

"In 117 *deaths*, inguinal bore the proportion to femoral as 44·4 per cent. to 55·5, only 11·1 per cent. being the difference *against* femoral hernia. But as the difference in recoveries was 27·7 per cent., it is clear that inguinal hernia is more fatal than femoral by 16·6 per cent."

We have room only for the conclusions drawn from the various analyses, and presented in the following form:

"1. That *inguinal* hernia is more common than femoral by 77·7 per cent.

"2. That *inguinal* hernia most frequently commences between 20 and 40 years of age, and *femoral* between 50 and 70.

"3. That the average duration of inguinal hernia prior to its strangulation is 20 years, of femoral 11.

"4. That inguinal hernia most frequently becomes strangulated before 50 years of age, and femoral after this time.

"5. That the average age of persons with strangulated inguinal hernia is 43, of femoral 55.

"6. That femoral hernia requires operation 25·2 per cent. more frequently than inguinal, success in its reduction by the taxis being less frequent.

"7. That, after operation, inguinal hernia is more fatal than femoral by 16·6 per cent.

"8. That 22 per cent of cases requiring operation are recent, and are strangulated on the first descent.

"9. That three-fourths of these recent cases are *femoral.*

"10. That the average period of strangulation in fatal cases of inguinal hernia is 50½ hours, of femoral 76½.

"11. That half the fatal cases of hernia die within 48 hours after the operation, and four-fifths within the first week.

"12. That three-fourths of the cases which refuse to rally after operation, are *femoral.*

"13. That collapse and death after a copious motion is by no means a rare occurrence.

"14. That artificial anus much more frequently follows *femoral* than inguinal hernia.

"15. That the cause of death in cases of artificial anus is generally exhaustion; that death occurs more rapidly when the intestine is slit open at the time of operation, than when the opening in the bowel naturally follows its return into the abdomen.

"16. That sudden collapse and death occasionally occur in cases of hernia which have progressed favorably for many days, without any *local* cause.

"17. That in about 69 per cent. of fatal cases, peritonitis exists sufficient to produce death; that is to say, lymph in some of its forms is generally effused.

"18. That in nine-tenths of the cases of hernia, the *ileum* is the portion of bowel strangulated; and in three-fourths of these it is part of the last two feet.

"19. That as a rule, the strangulated bowel, when returned, rests or is fixed by adhesions at the mouth of the sac.

"20. That in *all* cases of gangrenous bowel, the affected portion will be found at, if not adherent to, the mouth of the sac.

"21. That femoral hernia is much more frequently associated with gangrenous bowel than inguinal.

"22. That ulceration at the line of stricture is more frequent in inguinal hernia; although the sulcated condition of the bowel is as common in femoral as in inguinal.

"23. That fecal extravasation, if not produced by ruptured bowel from the taxis, generally follows ulceration at the line of stricture, and is consequently generally found in inguinal hernia.

"24. That ruptured bowel from the taxis is most frequent in femoral hernia.

"25. That fecal extravasation does not necessarily follow rupture of intestine by the taxis.

"26. That fecal extravasation does not occur so often as 50 per cent. in cases of rupture or perforation of the bowel.

The paper then closes with some very valuable practical deductions, for which we must refer the reader to the report itself, as one that will well repay him for its careful study.

Article Fifth is an interesting, and instructive paper, by Henry Oldham, M.D., on "Concealed Accidental Uterine Hemorrhage," in which three cases are given in full, with practical conclusions. One of the cases is illustrated by a lithographic print of the placenta, with its uterine surface hollowed out by the pressure of the coagulated blood.

In the Sixth Article, we have an elaborate and instructive view, by Dr. Wilks, of 45 cases of "Lardaceous Disease, and some Allied Affections," with remarks in which the distinguishing characteristics of this form of degeneration generally, and in different organs, are described at length and with great precision.

The closing paragraphs of this paper, are devoted to a description of a peculiar enlargement of the lymphatic glands existing in association with lardaceous spleen, which was noticed formerly by Dr. Hodgkin, and referred to by Dr. Bright, but which had recently attracted the attention of Dr. Wilks.

Succeeding the article just noticed is another (VII), by the same reporter, in which the results are given of the post-mortem examination of twelve fatal cases of burns.

The object of these examinations was to test the frequency of the occurrence of ulceration of the duodenum in such cases, as stated by Mr. Curling in his paper (Medico-Chirurg. Trans. vol. 25), on the subject. According to Dr. Wilks, the few opportunities afforded him of making examinations, had not corroborated Mr. Curling's experience; but he determined to subject the matter to a more careful trial, and, for the purpose, noted twelve cases in which death had supervened at various intervals of from nine days to seven weeks.

"It will be seen that in no case," says the reporter, "was there any disease discoverable in the duodenum after death, and that as regards the remaining part of the alimentary canal, in one case only was a slight diphtheritic condition of the colon found, and in another an erosion of the stomach." The latter form of ulcer is far from uncommon, being frequently met with in those dying from heart disease and other causes. In none of these cases, moreover, were there any symptoms denoting severe lesion of the intestinal canal during life; and the same fact is true of all the other fatal cases (25) which were not examined, as well as all those which recovered during the above named period (eighteen months).

Another article (VIII), by Dr. Wilks, completes the contribution of the able editor to this volume. It is entitled a "Brief Report of the Post-Mortem Examination of the Cases of Fever which have died in the Hospital during the last two years," and is in continuation of the account given in the last volume of the Reports, of all the cases of fever up to the autumn of 1854.

From this paper we shall make only one short extract:

"With reference to the question of specific differences between typhus and typhoid which was entered upon in the last volume, we may state that the greater number of cases being of the typhus kind, with the presence of a mulberry rash, the distinction has been well marked and the diagnosis easy; but in some of the milder forms the symptoms were considered to be not sufficiently distinctive to warrant their separation into species. Whatever difficulties, however, may have existed in the attempt to dissever the resemblances which may have been present during life, no such difficulties were found after death; for then a clear distinction was apparent between the two forms. In one class of cases no disease whatever was found in the intestine, and in the other a very remarkable condition and one altogether characteristic. There has been no intermediate state or point of transition between them, but a healthy condition on the one hand and a marked disease on the other. In most of the cases the symptoms of each class were distinct, and led to the knowledge of the appearances found after death, thus tending, as we believe, to make a clear separation between them. Leaving, however, for the present, the symptoms which existed during life, and regarding merely the appearances found after death, we cannot but conclude that we are looking upon diseases of altogether different kind." (pp. 137, 138.)

The next two articles (IX and X), are by Dr. William Gull, the first being a very well-prepared report of sixteen cases of paraplegia from tumors compressing the spinal cord, and the second a short memoir on the parasitical vegetable nature of ptyriasis versicolor.

The difficulty of diagnosis in paraplegia, the vagueness of the early symptoms, and the rarity of tumors of the cord, or its membranes, as a cause of paraplegia, render these admirably recorded and considered observations especially important and interesting. Their study in relation to the views of Brown-Sequard, and others, as to the functions of the different portions of the cord, would be interesting, but we have room only to notice some of the general considerations with which the special histories of the cases are prefaced.

Except the scrofulous deposits, tumors of the cord are most frequently seated in the loose tissue under the visceral layer of the arachnoid, or grow from the inner surface of the dura mater. They are not malignant as generally supposed, but *fibro-nuclear*, or *fibro-cellular* cancerous affections of the cord and its membranes, being extremely rare as a primary disease.

Pain appears to be most frequently "a prominent and characteristic symptom," and very variable in its character. Usually it is referred to the back, and more or less correctly indicates the seat of the disease, from which it radiates in the direction of the nerves whose roots are invaded; and even when there is no actual pain, there may be other disorders of *sensation* of various kinds. Next to pain, as an attendant on these affections, is the frequency of muscular contractions in the affected limbs, followed, as the case progresses, by flexion and rigidity, and attended by a great susceptibility to the excito-motor stimulus. "These phenomena are most apparent when the cord is merely stretched or compressed, and where no other change has occurred in it beyond atrophy, the communication with the brain being at the same time not entirely destroyed. If there be inflammatory softening of the substance of the cord, the then characteristic symptoms may be absent, as they are also in compression of the cord from fracture, when its structure is bruised and softened. Spasmodic contractions of the muscles of the extremities occur, whether the fibres of the cord are compressed by tumors on its surface, or stretched by tubercle deposited within it. At one stage of a case there may be rigid extension, which may be gradually followed as the case progresses by as rigid flexion, though the muscles at the same time may become atrophied and flaccid."

The practical importance of attending to the vagueness of the early symptoms in paraplegia is well illustrated by the reporter. "In the first case here recorded, the early symptoms, cough and slight dyspnœa, and some pain in the back and shoulders, were referred to tubercular disease of the lungs. In the second, the spasmodic action of the limbs was so great that for a time the case was regarded as one of hysteria. In fine, the symptoms of neuralgia, hysteria, lumbago, rheumatism, phthisis, colic, renal calculus, pleuritic and hepatic affections, may rise like so many phantoms, to delude us at the outset of most paraplegic affections, and the errors they are apt to lead to can be avoided only by a rigid inquiry."

Article XI, is a suggestive one by Dr. Habershon, entitled "Observations on the Abdominal Sympathetic Nerve." The chief point in this paper is the account given of the arrangement and relations of the semilunar ganglion and the diaphragmatic ganglion, with the union in the latter of the phrenic, pneumogastric and splanchnic nerves. This union, which is well displayed in an accompanying print, he justly dwells upon as particularly interesting.

"The use of the diaphragmatic ganglion appears to bring the diaphragm into intimate relation in its action with the abdominal viscera, and to unite the digestive and respiratory and cardiac centres of the sympathetic nerve. The pneumogastric and phrenic are the most important nerves of respiration; and here they are brought into close connection with the splanchnic, the great nerve of digestion, and send branches to the liver, the largest of the abdominal glands." (pp. 201, 202.)

This idea is sustained with an excellent practical review of the pathological phenomena, produced by the sympathetic action of their different centres, under the disturbing influence of disease in any one of them. We should be glad to lay it before our readers as a most useful lesson.

We must hasten on, however, to the remaining papers, and shall be obliged, under the restriction of rapidly decreasing space, to pass more briefly over much that is worthy of attention in the latter half of the volume.

Dr. Habershon is the author also of the article (XII) next in order. It is "On Dysphagia, Illustrated by some cases of Diseases of the Œsophagus and Pharynx." No one who has encountered in his practice any of these distressing forms of disease, can fail to appreciate the value of the experience which is so well and faithfully presented in this paper.

The causes of dysphagia illustrated as chiefly operative in the cases selected by Dr. Habershon, are given as follows:

"I. From disease of the tonsils or palate.

"II. From inflammation of the cellular tissue of the pharynx or œsophagus.

"III. From disease of the laryngeal cartilages, or epiglottis.

"IV. From functional or spasmodic stricture of the œsophagus or pharynx, as in hysteria, hydrophobia, &c.

"V. From paralysis of the muscles.

"VI. From acute inflammation of the mucous membrane.

"VII. From mechanical injury or poisons.

"VIII. From structural obstruction to the œsophagus, as—

"1. Constriction;

"2. Ulcerations, sometimes communicating with the larynx;

"3. Cancerous disease;

"4. Obstruction from the pressure of aneurismal or other tumors." (pp. 206, 207.)

A useful and opportune article next follows, by Mr. France, on the use of atropine in iritis, with an appendix containing two cases of artificial pupil, successfully established by operation.

The remarks on atropine in iritis conclusively combat the mischievous fallacy, recently started by respectable authority, which opposes, as unnecessary and injurious, the resort to belladonna or its alkaloid, for the purpose of dilating the pupil during the acute stage of iritis.

We fully indorse, on the authority of considerable public and private experience, the summary of doctrine with which his article concludes.

"1. On plain anatomical grounds, cohesion of the iris and capsule is less to be apprehended; and, when happening, is less injurious, with an expanded than a contracted state of pupil.

"2. The means of producing the former state should, therefore, always be employed in iritis; for though, when the complaint has reached a certain stage, the reaction of the pupil may be suspended; yet, in the majority of instances, this stage has not supervened when cases first apply.

"3. Should the tentative use of belladonna not attain the desired object, its effect is for the time simply negative—is not injurious.*

"4. Perseverance with belladonna or atropine, even when no immediate effect results, has this recommendation,—that it takes advantage, at the earliest period, of the returning mobility of the iris, when inflammation abates.

"5. During the convalescing stage, the detachment of adhesions is greatly promoted by the use of these agents, and, when accomplished, is a material and unmingled gain." (p. 240.)

Of the remaining six articles yet unnoticed, two are original and instructive essays by Mr. Odling, on the "Alkaline Emanation from Sewers and Cesspools," and on the "Detection of Antimony for Medico-Legal Purposes;" two are by Dr. Pavy, and two by Dr. Taylor.

* "Exceptional examples, wherein augmented pain follows the application, scarcely impair the universality of this rule; as the inconvenience rarely arises, and subsides on the discontinuance of the remedy."

The first of Dr. Taylor's contributions is his well-known and masterly review of the Palmer evidence, in relation to the question of poisoning by strychnia. This has already circulated widely in a separate form, and as we hope to examine it at length on another occasion, we shall say nothing more about it here.

The second article of Dr. Taylor's is occupied with an Analysis of the Water of the Great Geyser, Iceland; from which it appears that the only alkaline base contained in this water was soda. "This was associated with carbonic acid, chlorine, sulphuric and silicic acids; and the mineral constituents of the water, besides a minute trace of oxide of iron, were chloride of sodium, carbonate of soda, sulphate of soda, and silica, the last being the preponderating mineral ingredient."

Dr. Pavy's first paper (Art. XVII) is a very interesting experimental essay "On the Gastric Juice as a Solvent of the Tissues of Living Animals;" in which, by subjecting, through a fistulous orifice in the stomach of a dog, the ear of a live rabbit to the action of the gastric juice, he demonstrates the solvent action of this juice upon the living tissues of warm-blooded animals, as well as upon those of the frog and other lower grades of animal life.

Dr. Pavy's second contribution is entitled "Remarks on the Physiological Effects of Strychnia and the Woorali Poison." The subject is an interesting one, especially in view of the greater prevalence of strychnia poisoning, and of the recently all-absorbing inquiry on the Rugeley cases, and the subsequent trial, so ably discussed by Dr. Taylor.

In our analysis of Dr. Pavy's essay, we have availed ourselves of the critical assistance of a distinguished physiological friend and experienced vivisector, and shall append his notes.

The principal object of the paper is to show that these two poisons have an entirely different action, one of them exciting and leading to muscular action, the other depressing and leading to muscular paralysis; but that neither has any direct influence upon the heart.

The apparently exciting action of strychnia upon the spinal cord is well known, but it is questioned whether it acts directly upon the heart or not. In the frog, says Dr. Pavy, if you open the thorax, after poisoning with strychnia, when the animal appears perfectly dead, you find the heart still beating with its ordinary regularity, as will even be the case for several hours afterwards.* If strychnia is put directly on the heart, the frog dies from tetanus, but the heart continues to beat for more than two hours. Dr.

* Dr. Pavy seems not to be aware that the same observation had already been made by M. Ad. Moreau, in a paper on the action of poisons on the heart, read in November, 1855, at the Paris Société de Biologie. (*See Comptes Rendus et Mém. de la Soc. de Biol.*, 2d series, vol. ii, p. 171.)

Harley has obtained different results, because he employed strychnia dissolved in a menstruum (as acetic or hydrochloric acid), which soon causes the pulsations of the heart to cease, giving it a shrunken or contracted appearance.

Dr. Harley thinks that strychnia does not destroy life in producing asphyxia from spasm of the respiratory muscles, because he did not succeed in maintaining life by means of the artificial respiration he employed. Dr. Pavy says that, in his own experiments he has found it impossible to succeed in performing artificial respiration, unless the influence of the strychnized muscles be removed, by opening the chest. He relates two experiments, one upon a dog, the other upon a rabbit, which show that if the respiration be thus sustained, after poisoning with strychnia, the action of the heart will continue. In the dog, when the chest was opened, after apparent death, the heart was beating most feebly and irregularly; but on performing artificial respiration, its action became rapid and strong, and was thus kept up for twenty minutes. When the respiration was interrupted, the heart soon ceased to beat. The experiment upon the rabbit gave similar results.*

Dr. Pavy has performed some other experiments, which show that the heart, in animals killed by strychnia, has its cavities in the same condition as in cases of asphyxia, *i. e.*, the right side full, and the left empty. All his experiments lead him to think that strychnia kills, in producing asphyxia, by the spasm of the respiratory muscles.

The woorali poison acts in producing paralysis. Dr. Pavy describes an experiment to show that it destroys nervous power, and not the vital property of muscles, and from that draws the conclusion, that muscular irritability is independent of nervous action. Besides, Dr. Pavy shows that the heart's action is not directly affected by this poison.†—(*Guy's Hosp. Reports*, vol. ii, p. 408–16, 1856.)

* Dr. Pavy says that the galvanization of the pneumogastric nerve produces a spasm of the heart, just as the galvanization of any muscular nerve causes contraction in the muscle to which it is distributed. We doubt this fact. The heart's action is truly stopped when the vagus nerve is galvanized, but there is no spasm then produced; the heart, on the contrary, dilates by the afflux of blood in its cavities, as the brothers Weber have found. Perhaps Dr. Pavy had applied one of the conductors on the heart, while the other was on the pneumogastric. In such a case, as it has been pointed out by Dr. Brown-Sequard (Gazette Med. de Paris, 1850, p. 332), the heart contracts spasmodically during the galvanization.

† Dr. Pavy seems to be ignorant that the results of his experiments had been already obtained by other experimenters, among whom we will name Sir B. Brodie, Prof. Cl. Bernard, and Koelliker, who have even gone further than Dr. Pavy. Sir B. Brodie has found that if insufflation is continued long enough after poisoning

